# Fatal Errors

**Published:** 1882-10

**Authors:** Ad. Lippe

**Affiliations:** Philadelphia


### FATAL ERRORS.

AD. LIPPE, M. D., PHILADELPHIA.

At the close of the last session of the American Institute the following resolution was adopted:

> "*Resolved*, That it is the sense of the American Institute that no physician can properly sustain the responsibilities, or fulfill all the duties of his professional relations, unless he enjoys absolute freedom of medical opinion, and unrestricted liberty of professional action, as provided for in the Code of Ethics of this Institute."

What can this resolution really mean? As we became a member of the Institute at its second meeting, we may be supposed to know the aims of that Society. *Then* all the members held it to be incumbent upon its members to practice Homœopathy, as promulgated by its founder. We united to develop our knowledge by augmentations to our materia medica, that we might the better apply the only law of cure known to the healing-art. We enjoyed then, as now, absolute freedom of medical opinion; no law of this free land could prevent any one from either joining or leaving the Institute, provided he was, when he joined it, possessed of the requisite knowledge of medicine, and especially of Homœopathy. He could, if he found the healing-art called Homœopathy inadequate, when he tried to properly sustain the responsibilities or fulfill all the duties of his professional relations, leave the Institute at pleasure, all of which covered the unrestricted liberty of professional action. If such was the true meaning of this resolution, it was obviously unnecessary to offer or to pass it.

What can this resolution really mean? The learned compiler of so much, almost incomprehensible bombast, now kindly tells us what he did really mean—*vide Hahnemannian Monthly*, Sept. 1st, 1882, p. 560, and there and then he enlightens us thus: "Before the resolution was offered it was shown to several members of the Institute, each and all of whom at once understood it to refer to the question which now divides and distracts the allopathic denomination, viz.: the consultation question!"

What a revelation this is! For the sake of everything appertaining to language, we cannot see a syllable in this resolution touching either the question of consultation or recognition, nor are we aware of any—even the slightest—attempt made in behalf of the Institute forbidding any such consultations! Besides all this, the allopaths have very wisely settled the distracting question, and we have nothing to do with it. As the resolution has nothing whatever to do with "consultations," and as we must take it for granted that the chosen few members of the Institute did see said resolution "after dinner," we may now be permitted to put our own interpretation on this strange language. The gentleman who offered said resolution once delivered himself of this remark before the Philadelphia Homœopathic Medical Society—"I have a weakness for quinine; if I could get along without quinine, as some claim to do, I could get along without Homœopathy altogether," which might be put into more explicit language—"Homœopathy teaches the administration of quinine in massive doses for the cure of intermittent fever, as I understand it, and those who claim to get along without it are not homœopaths, as I understand it. No such Homœopathy without quinine for me." As Homœopathy is an exclusive system of therapeutics, as it teaches that all dynamic and all curable, not surgical, cases of disease (even if caused by mechanical injuries) are amenable under the Law of the Similars, excluding necessarily the palliative treatment of the allopathic school, and as it is evident this is not palatable to a learned gentleman who seems not to know that Homœopathy always rejected the ever pernicious use of crude drugs, quinine included, this resolution means nothing more or less than this: "I am a homœopath, but claim absolute freedom of medical opinion and unrestricted liberty of professional action—

*i. e.*, to give crude doses of quinine and morphia myself whenever I please, and express this my opinion freely without restriction." These are the prerogatives of the eclectics. As a logical sequence, the next resolution offered the Institute should read: "Whereas, the fatal error was committed of passing the above resolution last year, at the close of the session, we now apply the 'proper' remedy, and strike out from the title of our Association, founded by the early pioneers, the name Homœopathy, and insert in the place of it—eclecticism." That *is* the true inwardness of said resolution.

